# Cardiovascular responses produced by resistin injected into paraventricular nucleus mediated by the glutamatergic and CRFergic transmissions within rostral ventrolateral medulla

**DOI:** 10.22038/IJBMS.2019.40316.9547

**Published:** 2020-03

**Authors:** Abolfazl Akbari, Gholamali Jelodar

**Affiliations:** 1 Department of Physiology, School of Veterinary Medicine, Shiraz University, Shiraz, Iran

**Keywords:** Angiotensin II, Arterial pressure, Corticotrophin-releasing – hormone, Heart rate, L-Glutamate, Paraventricular hypothalamic – nucleus, Resistin

## Abstract

**Objective(s):**

Resistin, as a 12.5 kDa cysteine-rich polypeptide, is expressed in hypothalamus and regulates sympathetic nerve activity. It is associated with obesity, metabolic syndrome and cardiovascular diseases. In this study, we investigated the neural pathway of cardiovascular responses induced by injection of resistin into paraventricular nucleus (PVN) with rostral ventrolateral medulla (RVLM).

**Materials and Methods:**

Adult male rats were anesthetized with urethane (1.4 g/kg intraperitoneally). Resistin (3 µg/1 µl/rat) was first injected into PVN, and the glutamatergic, corticotrophin-releasing factor (CRF)-ergic and angiotensinogenic transmission was inhibited by injecting of their antagonist in RVLM. Arterial pressure (AP) and heart rate (HR) were monitored before and after the injection.

**Results:**

The results showed that resistin injection into PVN significantly increased AP and HR compared to control group and prior to its injection (*P*<0.05). Injection of AP5 ((2R)-amino-5-phosphonovaleric acid; (2R)-amino-5-phosphonopentanoate) (50 nM/rat), losartan (10 nM/rat) and astressin (50 nM/rat) into RVLM reduced cardiovascular responses produced by injected resistin into PVN. Injection of AP5+losartan or astressin+losartan or astressin+AP5 into RVLM could significantly reduce cardiovascular responses produced by resistin compared to before injection (*P*<0.05). Furthermore, the depressor responses generated by AP5+losartan injected into RVLM were significantly stronger than the depressor responses generated by AP5+astressin and/or astressin+losartan injected into RVLM (*P*<0.05).

**Conclusion:**

It can be concluded that glutamatergic and CRFergic transmissions have crucial contribution to cardiovascular responses produced by resistin. The results provided new and potentially important insight regarding neural transmission when the plasma level of resistin increases; this reveals the role of resistin in cardiovascular responses such as metabolic syndrome and hypertension.

## Introduction

It is well-known that sympathoexcitatory neurons of rostral ventrolateral medulla (RVLM) are responsible for producing sympathetic vasomotor tone and play an important role in tonic and reflex control of cardiovascular responses (1-4). Paraventricular hypothalamic nucleus (PVN) is one of the supramedullary nuclei of the brain that, like RVLM, plays an important role in controlling basal cardiovascular responses (4). This nucleus has several neuronal populations, which are involved in generating physiological response to energetic challenges (5), environmental stress and cardiovascular diseases (6). PVN performs its cardiovascular actions by sending axonal projections to RVLM or to sympathetic preganglionic neurons in the spinal intermediolateral cell column (IML), and synthesizes, storages and releases vasopressin (7). The neural projection from the PVN to the RVLM and to the IML of the spinal cord was predominantly unilateral (4, 6, 8). It was also reported that more than two-thirds of projections from PVN to RVLM is unilaterally (8, 9). The neurons of the PVN projecting to the RVLM are mainly parvocellular neurons (8), which control the baseline arterial pressure (AP) and heart rate (HR) (4). The PVN axonal projections to RVLM are more prominent than other projections (10). It has also been reported that the number of PVN neurons projecting to RVLM is seven-fold greater than the number of PVN neurons projecting to spinal cord (8). Reports have also shown that more than 30% of PVN neurons projecting to RVLM release corticotrophin-releasing hormone or factor (CRH/ CRF) (11), which generate cardiovascular responses and renal sympathetic nerve activity (SNA) (12, 13). Moreover, glutamatergic and angiotensinogenic transmissions are involved in generating pressor response within RVLM arising from PVN (13-15). These results indicate that neurotransmitters that mediate the activity of PVN-RVLM neurons have the potential to influence basal resting sympathetic tone, and can be considered as one of the therapeutic targets in diseases like heart failure (HF), metabolic syndrome, hypertension and other diseases associated with chronic sympathetic hyperactivity. 

Resistin as a 12.5 kDa cysteine-rich protein is expressed primarily in rodent white adipocytes (16) and human macrophages (17, 18). Moreover, resistin is expressed in different brain regions (19-21), mammary gland (22) and brown adipocytes (23). The expression of resistin is decreased by tumor necrosis factor α (24) and β-adrenergic agonists (24), and increased by androgens (25) and in a nutritional-, age- and gender-specific manner (26). The plasma levels of resistin have a positive correlation with HF (27, 28), obesity and metabolic syndromes (29) in which the activity of sympathetic nerve increases (30, 31). In addition, it has been suggested that resistin modulates the function of sympathetic neurons (32). Injection of resistin into lateral ventricle also increases SNA (33) that this effect may be mediated by phosphatidylinositol 3-kinase (34). Resistin, like leptin, can easily pass blood-brain barrier (BBB) under physiological and pathophysiological conditions via simple diffusion or other transport systems and can affect central nervous system (CNS) functions and subsequently, alter some metabolic activities in peripheral organs (34, 35). Resistin is widely expressed (mRNA and protein) in pituitary (26) and hypothalamus (20) with an important role in regulating food intake, energy homeostasis (21) and cardiovascular activities (36). Recently, in a pilot study with the aim of discovering cardiovascular responses of resistin and its optimal dosage, we found out that resistin injection (1,3 and 5 µg/rat) into PVN, in a dose-dependent manner, increases AP and HR (36). This finding definitely indicates that resistin-sensitive neurons within the PVN that modulate cardiovascular responses have resistin receptors. Moreover it was reported that Toll-like receptor 4 (TLR4) acts as resistin receptors in hypothalamus (37). Although it has not been known which parts of the PVN are expressed in resistin receptors, we indicated that cardiovascular responses generated by resistin into PVN might be modulated via collateral projections to neurons of RVLM by glutamatergic transmission (Unpublished). It is likely that other neuronal transmission such as CRFergic and angiotensinogenic mechanisms and PVN neurons projecting to the spinal cord may play a role in transmitting these responses. Therefore, the aim of this study was to determine the neural pathway of cardiovascular responses induced by resistin injection into the parvocellular neurons of PVN with sympathoexcitatory neurons within RVLM. 

## Materials and Methods

***General procedures***

Adult male Sprague-Dawley rats (280-320 g, 9-10 weeks old), which were colony-bred in the Animal House Center of Shiraz, Iran, were housed in animal room under controlled lighting (12 hr light: 12 hr darkness) and temperature (20±2 ^°^C) with free access to pelleted food (formulated and made by Javaneh Khorasan Company, Iran) and tap water. All investigations conducted in this study were in accordance with the “Guiding Principles for the Care and Use of Research Animals” approved by Shiraz University.

***General surgical preparation and cardiovascular response***

All animals were anesthetized by urethane (1.4 g/kg intraperitoneally). The paw pinch reflex was used to assess the depth of anesthesia. Continuous cardiovascular response measurements were performed directly using polyethylene catheters (PE-50) inserted into the left femoral artery. Polyethylene catheter was filled with heparinized saline and was connected to a pressure transducer (Grass Instrument Company, USA). The HR and AP were continuously recorded by a Grass polygraph (Model 7D Polygraph, Grass Instrument Co., USA) and a computer program, which was provided in this laboratory and by a digital electrocardiography (ECG) recorder (Suzuken Kenz, ECG 110**, **Suzuken Co., Japan). Body temperature was monitored by rectal temperature and maintained in the range of 37-38 ^°^C. The animals were placed in prone position in a Stereotaxic apparatus (Stoelting Co., USA) and a small hole was drilled through the parietal bone over PVN or RVLM. Stereotaxic coordination for the injection sites in PVN and RVLM was selected from the Rat Brain Atlas of Paxinos and Watson (2005) (38). Coordination of PVN was as follows: 7.44 mm rostral to the interaural line, right+0.5-0.7 mm from the medial suture and 7.8 mm deep from the bregma. Coordination of RVLM was as follows: 1.8-2.2 mm rostral to the caudal tip of the area postrema, 1.8 –2.2 mm lateral to the midline, and 3.0 –3.5 mm ventral to the dorsal surface of the medulla. Following the insertion of the catheter in the left femoral artery and stereotaxic, there was a waiting period of 20-25 minutes before measuring cardiovascular variables. AP and HR were recorded and assessed for a 15-30-minute period before injections, and a 90-120-minute period after the injection of resistin into PVN and a final 45-60-minute period after the injection of other drugs into RVLM.

***Experimental protocols***

This study aimed to evaluate the effects of resistin (3 µg/1 µl/rat, Phoenix Pharmaceuticals Inc., Karlsruhe, Germany) injection into PVN parvocellular neurons on cardiovascular response and its neural transmission with RVLM. The rats were randomly allocated in eleven groups (10 experimental and 1 control) (n=8) as follows:

1) The control group; All of the procedures were exactly the same as in the experimental groups, but instead of the drug, the vehicle (saline, 0.1 µl/rat) was injected into PVN or RVLM.

2) The experimental group 1: Resistin (3 µg/rat) was injected into PVN and normal saline (1 µl/rat) was injected into RVLM. 

3) The experimental group 2: Normal saline was injected into PVN and L-glutamate (50 nM/rat) was injected into RVLM. 

4) The experimental group 3: Resistin was injected into PVN and AP5 ((2*R*)-amino-5-phosphonovaleric acid; (2*R*)-amino-5-phosphonopentanoate), a selective *N*-methyl-D-aspartate receptor (NMDA) receptor antagonist (50 nM/rat), was injected into RVLM. 

5) The experimental group 4: Normal saline was injected into PVN and angiotensin II (ANG II) (100 pM/rat) was injected into RVLM. 

6) The experimental group 5: Resistin was injected into PVN and losartan, an ANG II receptor type 1 (AT1) receptor antagonist (10 nM/rat), was injected into RVLM. 

7) The experimental group 6: Normal saline was injected into PVN and CRF (50 nM/rat) was injected into RVLM. 8) The experimental group 7: Resistin was injected into PVN and astressin, an antagonist of CRF receptor (10 pM/rat), was injected into RVLM. 

9) The experimental group8: Resistin was injected into PVN and AP5 and CRF (0.1 µl/rat) were injected into RVLM. 

10) The experimental group9: Resistin was injected into PVN and AP5 and losartan (0.1 µl/rat) were injected into RVLM. 

11) The experimental group10: Resistin was injected into PVN and astressin along with losartan (0.1 µl/rat) were injected into RVLM. 

12) Injections of resistin and saline were performed into the right side of PVN and RVLM unilaterally. All injections were performed in the same volume (0.1 µl). 

***Injection of drugs***

The injections of recombinant murine resistin, normal saline and other drugs (Sigma-Aldrich) were made by pressure in the right side of PVN and RVLM using a hand-driven 1 μl syringe (KH7001; Hamilton, Reno, NV, USA) connected to a 33 gauge needle by PE-10 tubing. Injections were performed in a volume of 0.1 μl in PVN or RVLM. After the injection, the needle was kept within the guide cannula for 1 min.

***Histological verification***

**Figure 1:**
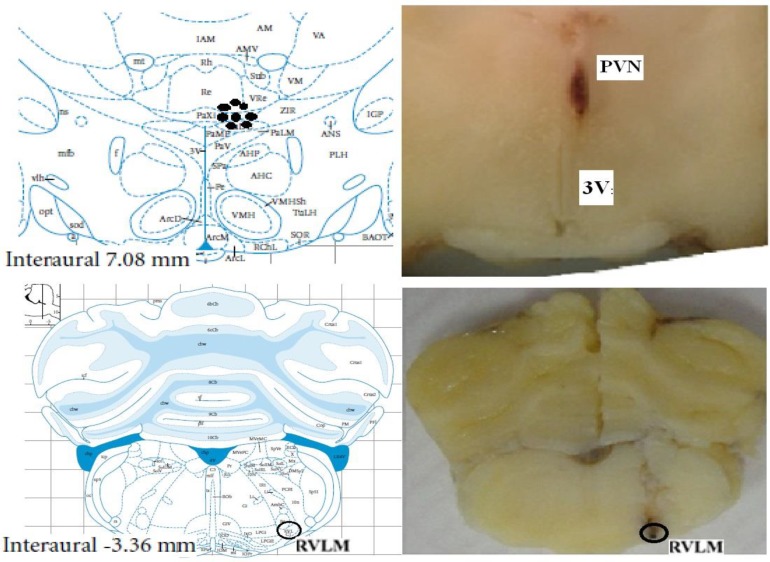
Diagrammatic representation is based on rat brain atlas of Paxinos & Watson (2005), which shows the dispersion of the injection sites among the experiments. PVN: Paraventricular Nucleus 3V: third ventricle, RVLM: Rostral ventrolateral medulla

At the end of each experiment in order to confirm the injected sites, 1 µl Evan’s Blue dye was injected as a marker into PVN and RVLM. After 10 minutes, 5 ml of 10% formalin was injected directly into the left ventricle. Then, the brain was removed and fixed in a 10% formalin solution for at least 3 weeks. After this period, serial sections were prepared from the tissues. The injection sites were confirmed according to Rat Brain Atlas of Paxinos and Watson (2005) (38) under the light microscope (Figure 1). 

***Data analysis***

The average values of AP and HR were measured before and during 90-120 minutes after each injection. The average values of these variables for a 20-minute period prior to injection were measured as the baseline levels. The maximum changes in AP and HR during 90-120 minutes after injections into PVN or RVLM were compared to before injection by paired t-test and or were compared to those after vehicle injection into the same region by the independent t-test. The other results were analyzed using two-way analysis of variance followed by *post hoc *Tukey’s multiple comparisons test for comparison between different treatment groups. A probability value *P*<0.05 was taken to indicate a statistically significant difference. All data are presented as mean± standard error of mean (±SEM). The data were analyzed by Statistical Package for Social Sciences (SPSS 16.0 for Windows). 

## Results

***The effect of resistin/normal saline injected into PVN and agonists or antagonists injected into RVLM on cardiovascular responses to detect neural connection between PVN and RVLM***

*Cardiovascular responses to normal saline (0.1 µl/rat) injected into PVN and RVLM*

**Figure 2:**
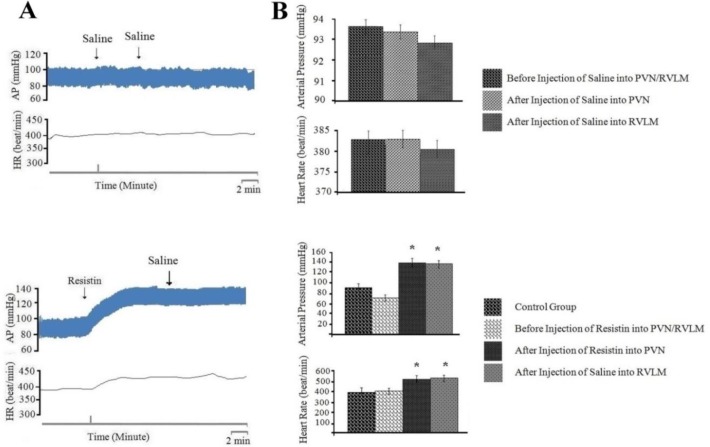
Cardiovascular responses (arterial pressure and heart rate) to saline (0.1 µl/rat) or resistin (3 µg/rat) injected into paraventricular nucleus (PVN), and saline (0.1 µl/rat) injected into rostral ventrolateral medulla (RVLM). A: Typical recording of arterial pressure (AP) and heart rate (HR) to saline or resistin injected into PVN and saline injected into RVLM. The vertical lines indicate the injection time. B: Mean ±standard error of mean (SEM) for changes in AP and HR before and after the injection of saline or resistin into PVN and saline into RVLM compared to control group (n=8). The upper right figure is related to the control group and is then compared to other groups. Asterisk shows the statistical difference between prior and after injection and with control group

Vehicle group: In this group, the baseline of AP and HR was 89±1 mmHg and 372±3 beats/min, respectively. The injection of normal saline (0.1 µl/rat) into PVN did not affect the AP (ΔAP=1.2±0.1 mm Hg) and HR (ΔHR=4±1 beats/min; n=8 rats). In addition, injection of normal saline (0.1 µl/rat) into RVLM had no effect on cardiovascular responses (Figure 2, *P*<0.05).

***The effect of normal saline (0.1 µl/rat) injection into RVLM on cardiovascular response generated by resistin (3 µg/rat) injection into PVN***

To study the effects of resistin on cardiovascular responses and to detect the neuronal transmission between PVN and RVLM that mediate these responses, 3 µg/rat of resistin was injected into PVN and then saline (0.1 µl/rat) was injected into RVLM; this group was considered as the sham group. In this group, the baseline of AP and HR was 84±12 mmHg and 392±25 beats/minute, respectively. The injection of resistin into PVN caused a significant increase in AP (ΔAP=73±12 mmHg) or HR (ΔHR=118±24 beats/min; n=8 rats). The time course of cardiovascular responses to resistin injection into PVN was very fast, so that in the first moments after injection of resistin the blood pressure began to rise and reached a peak after 1-2 minutes. Injection of normal saline into RVLM had no significant effect on AP and HR (Figure 2, *P*<0.05).

***Cardiovascular responses to normal saline (0.1 µl/rat) injected into PVN and L-glutamate (50 nM/rat) injected into RVLM***

**Figure 3:**
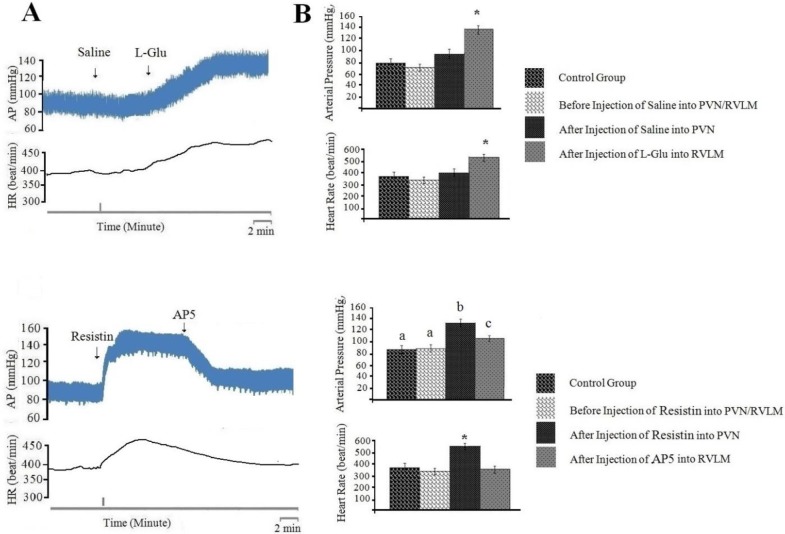
Cardiovascular responses (arterial pressure and heart rate) to saline (0.1 µl/rat) or resistin (3 µg/rat) injected into paraventricular nucleus (PVN) and AP5 ((2R)-amino-5-phosphonovaleric acid; (2R)-amino-5-phosphonopentanoate) (50 nM/rat) or L-glutamate (50 nM/rat) injected into rostral ventrolateral medulla (RVLM). A: Typical recording of arterial pressure (AP) and heart rate (HR) to saline or resistin injected into PVN and AP5 or L-glutamate injected into RVLM. The vertical lines indicate the injection time. B: Mean±standard error of mean (SEM) changes in AP and HR before and after the injection of saline or resistin into PVN and AP5 or L-glutamate into RVLM compared to control group (n=8). Asterisk and different small letters show the statistical difference between the time before and after the injection with control group

In this group, the baseline of AP and HR was 86±11 mmHg and 385±23 beats/min, respectively. Injection of normal saline (0.1 µl/rat) into PVN had no significant effects on AP and HR. However, the injection of L-glutamate into RVLM significantly increased AP (ΔAP=46±12 mm Hg) and HR (ΔHR=85±22 beats/min; n=8 rats) compared to pre-injection and control group (Figure 3, *P*<0.05). 

***The effects of AP5 (50*** ***nM/rat) injection into RVLM on cardiovascular response generated by resistin (3 µg/rat) injection into PVN***

In this group, the baseline of AP and HR was 88±10 mmHg and 387±20 beats/min, respectively. Injection of resistin (3 µg/rat) into PVN caused a significant pressor response on AP (ΔAP=87±10 mm Hg) and HR (ΔHR=86±18 beats/min; n=8 rats) compared to before injection and control group. Injection of AP5 into RVLM significantly decreased AP (ΔAP= -53±12 mm Hg) and HR (ΔHR= -85±22 beats/min; n=8 rats) compared to pre-injection (Figure 3, *P*<0.05). Figure 3A shows a typical recording of AP and HR to AP5 (50 nM/rat) injected into RVLM following resistin (3 µg/rat) injected into PVN. The analyzed data in Figure 3B indicated that pressor responses generated by resistin into PVN significantly reduce by AP5 injected into RVLM compared to before injection (Figure 3, *P*<0.05). 

***Injection of normal saline (0.1 µl/rat) into PVN had no effects on cardiovascular responses and angiotensin II (100 pM/rat) injected into RVLM produces cardiovascular responses***

**Figure 4:**
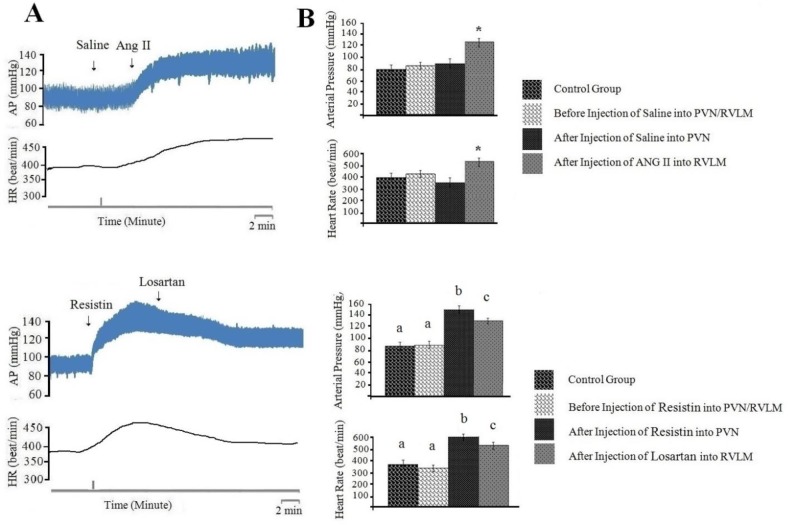
Cardiovascular responses (arterial pressure and heart rate) to saline (0.1 µl/rat) or resistin (3 µg/rat) injected into paraventricular nucleus (PVN) and losartan (10 nM/rat) or angiotensin II (ANG II) (100 pM/rat) injected into rostral ventrolateral medulla (RVLM). A: Typical recording of arterial pressure (AP) and heart rate (HR) to saline or resistin injected into PVN and losartan or ANG II injected into RVLM. Vertical lines indicate the injection time. B: Mean ± standard error of mean (SEM) changes in AP and HR before and after the injection of saline or resistin into PVN and losartan or ANG II into RVLM compared to control group (n=8). Asterisk and different small letters show the statistical difference between the time before and after the injection with control group

In fact, the main goal of this study was to identify neuronal pathways involved in the development of cardiovascular responses produced by resistin. Therefore, first cardiovascular responses generated by injection of angiotensin into RVLM were examined, then the effects of losartan (10 nM/rat) injected into RVLM on cardiovascular responses generated by resistin (3 µg/rat) into PVN were examined. In this study, the baseline of AP and HR was 81±10 mmHg and 394±25 beat/min, respectively. The results showed that the injection of normal saline (0.1 µl/rat) into PVN had no effects on AP (ΔAP=3±1 mm Hg) and HR (ΔHR=6±1beats/min; n=8) (Figure 4, *P*>0.05). However, injection of ANG II (100 pM/rat) into RVLM significantly increased arterial pressure (ΔAP=63±13 mmHg) and heart rate (ΔHR=85±13 beats/min; n=8 rats) compared to before injection and control group (Figure 4, *P*<0.05).

***Injection of losartan (10 nM/rat) into RVLM could reduce cardiovascular response generated by resistin (3 µg/rat) injection into PVN***

The baseline of AP and HR was 80±11 mmHg and 390±21 beats/min, respectively. Injection of resistin (3 µg/rat) into PVN significantly increased AP (ΔAP=70±11 mmHg) and HR (ΔHR=89±20 beats/min; n=8 rats). The results showed that injection of losartan (10 nM/rat) into RVLM significantly decreased cardiovascular responses generated by resistin (ΔAP=-23±8 mmHg, ΔHR=-61±11 beats/min; n=8 rats) compared to before injection (Figure 4, *P*<0.05). The changes of these responses were very slow with long-term effects, which lasted 45-60 minutes after the injection of losartan. The results also showed that the depressor response produced by losartan was lower than the depressor response produced by AP5 after resistin injection into PVN (*P*<0.05). 

***Injection of normal saline (0.1 µl/rat) into PVN had no effects on cardiovascular responses and CRF (50 nM/rat) injected into RVLM produces cardiovascular responses***

**Figure 5:**
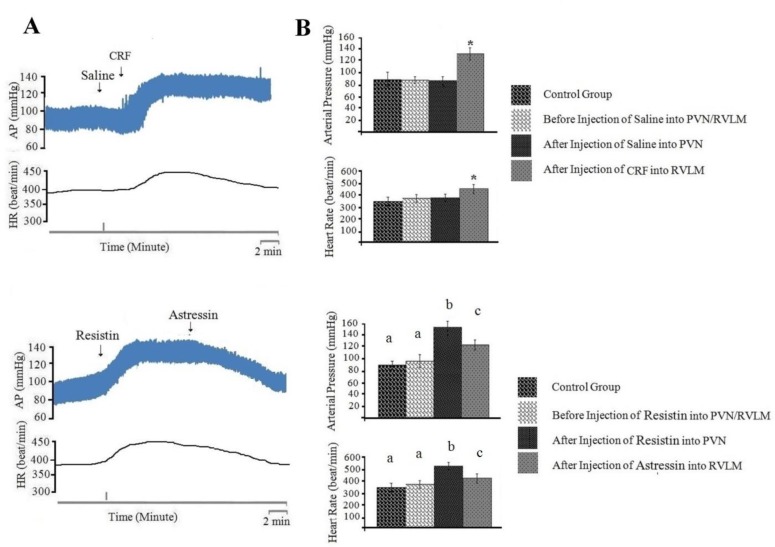
Cardiovascular responses (arterial pressure and heart rate) to normal saline (0.1 µl/rat) or resistin (3 µg/rat) injected into paraventricular nucleus (PVN) and corticotrophin-releasing factor (CRF) (10 nM/rat) or astressin (50 nM/rat) injected into rostral ventrolateral medulla (RVLM). A: Typical recording of arterial pressure (AP) and heart rate (HR) to saline or resistin injected into PVN and CRF or astressin injected into RVLM. The vertical lines indicate the injection time. B: Mean ± standard error of mean (SEM) changes in AP and HR before and after the injection of saline or resistin into PVN and CRF or astressin into RVLM compared to control group (n=8). Asterisk and different small letters show statistical difference between the time before and after injection with control group

The baseline of AP and HR was 83±12 mmHg and 364±17 beats/min, respectively. Injection of normal saline (0.1 µl/rat) into PVN did not affect AP (ΔAP=3±1 mm Hg) and HR (ΔHR=6±1 beats/min; n=8 rats). Injection of CRF into RVLM significantly increased AP (ΔAP=67±13 mm Hg) and HR (ΔHR=96±11 beats/min) compared to before injection (Figure 5, *P*<0.05).

***The effect of astressin*** ***(50 nM/rat) injection into RVLM on cardiovascular response generated by resistin (3 µg/rat) injection into PVN***

In this group, the baseline of AP and HR was 89±12 mmHg and 368±18 beats/min, respectively. Injection of resistin (3 µg/rat) into PVN caused a significant pressor response on AP (ΔAP=84±14 mm Hg) and HR (ΔHR=96±14 beats/min; n=8 rats), respectively compared to before injection and control group. Cardiovascular responses (ΔAP=-36±8 mmHg, ΔHR=-76±11 beats/min; n=8 rats) significantly decreased by injection of astressin (50 nM/rat) into RVLM compared to before injection (Figure 5, *P*<0.05). Figure 5A shows a typical recording of AP and HR to astressin (50 nM/rat) injected into RVLM following resistin (3 µg/rat) injected into PVN. The analyzed data in Figure 5B indicated that AP and HR significantly decreased (*P*<0.05) following astressin injection into RVLM compared to the time before the injection. 

***The effect of antagonists of glutamatergic, angiotensinogenic and CRFergic receptors injected into RVLM on cardiovascular responses induced by resistin injected into PVN and to discover neural transmission between PVN and RVLM***

**Figure 6:**
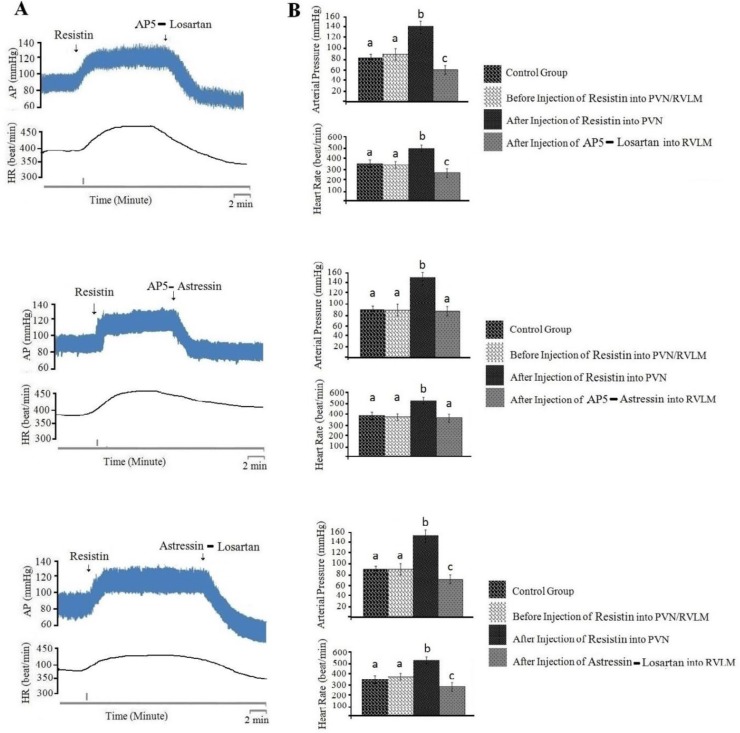
Cardiovascular responses (arterial pressure and heart rate) to resistin (3 µg/rat) injected into paraventricular nucleus (PVN) and antagonists of glutamatergic (AP5, 50 nM/rat), angiotensinogenic (losartan, 10 nM/rat) and CRFergic (astressin, 50 nM/rat) receptors injected into rostral ventrolateral medulla (RVLM). A: Typical recording of arterial pressure (AP) and heart rate (HR) to resistin injected into PVN and other drugs injected into RVLM. The vertical lines indicate the injection time. B: Mean±standard error of mean (SEM) changes in AP and HR before and after injection of resistin into PVN and other drugs into RVLM compared to control group (n=8). The small different alphbetics show the statistical difference between the time before and after the injection with the control group

**Figure 7:**
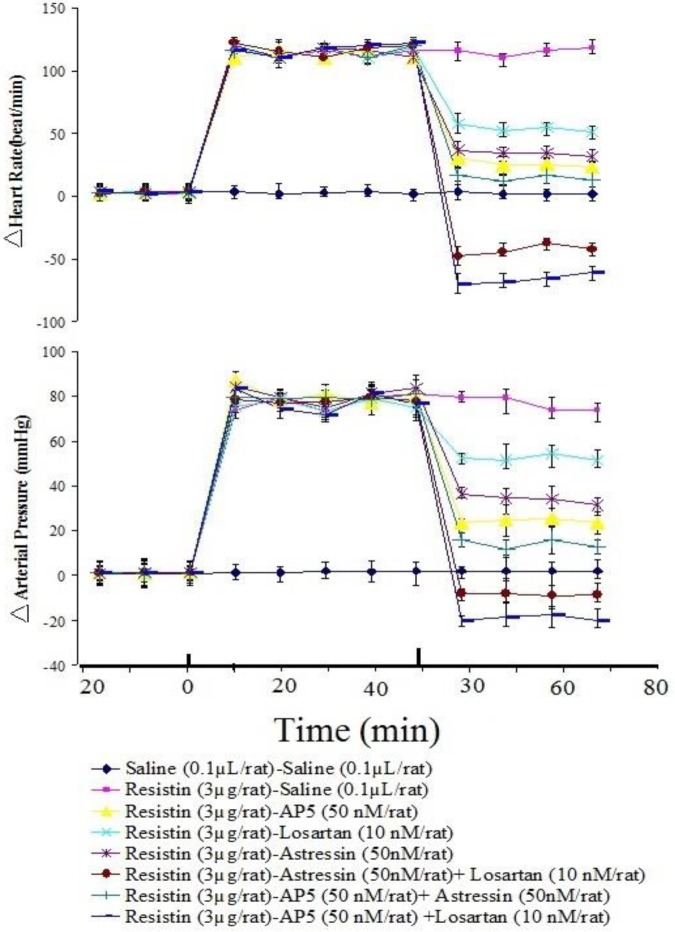
Time course for astressin, losartan and AP5 ((2R)-amino-5-phosphonovaleric acid; (2R)-amino-5-phosphonopentanoate) injection into rostral ventrolateral medulla (RVLM) following resistin injected in paraventricular nucleus (PVN) on HR and AP in urethane-anesthetized rats. The vertical lines indicate the injection time. In each case, the time zero represents the point at which injections were made. Significant difference was observed among groups from 5 min after injection of drugs and later. All values are represented as mean± standard error of mean (SEM)

To discover neuronal transmissions within RVLM that are responsible for producing the cardiovascular responses generated by injection of resistin into PVN, we studied the role of glutamatergic, angiotensinogenic and CRFergic transmissions. Figures 2-5 show the analyzed data and a typical recording of AP and HR in response to the antagonist injection of glutamatergic (AP5, 50 nM/rat), angiotensinogenic (losartan, 10 nM/rat) and CRFergic (astressin, 50 nM/rat) receptors in RVLM following resistin (3 µg/rat) injection into PVN. The results indicated that injection of resistin into PVN significantly increased AP and HR (*P*<0.05), and injection of each antagonists of glutamatergic, angiotensinegic and CRFergic receptors into RVLM significantly reduced cardiovascular responses produced by injection of resistin into PVN (Figure 2-5, *P*<0.05). Injection of antagonists of glutamatergic (AP5, 50 nM/rat) along with CRFergic (astressin, 50 nM/rat) receptors into RVLM significantly reduced cardiovascular responses produced by resistin injection into PVN compared to before injection (Figure 6, *P*<0.05). The results also showed that injection of antagonists of glutamatergic (AP5, 50 nM/rat) along with angiotensinogenic (losartan, 10 nM/rat) receptors into RVLM could significantly reduce cardiovascular responses produced by resistin injection into PVN compared to before injection (Figure 6, *P*<0.05). The results of this study also showed that astressin (50 nM/rat) along with losartan (10 nM/rat) injected into RVLM significantly reduced cardiovascular responses induced by resistin compared to before injection (Figure 6, *P*<0.05). The results obtained from AP5, astressin and losartan injected into RVLM showed that AP5 has the greatest inhibitory effects, and astressin has a higher inhibitory effect, in comparison with losartan, on cardiovascular responses generated by resistin (*P*<0.05, Figure 7). Statistical comparison of the three groups indicated that the depressor responses generated by AP5+losartan injected into RVLM were significantly stronger than the depressor responses generated by AP5+ astressin and/or astressin+ losartan injected into RVLM (*P*<0.05, Figure 7). The depressor responses generated by astressin +losartan injected into RVLM were stronger than the depressor responses generated by AP5+ astressin injected into RVLM (*P*<0.05, Figure 7). On the other hand, statistical analysis showed that the depressor responses generated by AP5+losartan, astressin+ losartan and AP5+ astressin injected into RVLM were significantly stronger than the depressor responses generated by AP5, losartan and astressin injected into RVLM (*P*<0.05, Figure 7). 

## Discussion

The results of this study indicated that injection of L-glutamate into RVLM could produce cardiovascular responses, while injection of AP5 into RVLM reduced cardiovascular responses produced by resistin injection into PVN, which is consistent with the results obtained by Yang and Coote (1998), and Zhou *et al.* (2006). Yang and Coote (1998) showed that injection of a NMDA receptor agonist into PVN increases neuronal activity in RVLM neurons and blood pressure, while these responses are inhibited by a glutamate receptor blocker in RVLM (39). Zhou *et al.* (2006) suggested that glutamate in RVLM acts as an excitatory neurotransmitter through NMDA and α-amino-3-hydroxy-5-methyl-4-isoxazolepropionic acid (AMPA) receptors (40). However, Xu *et al.* (2012) showed that PVN-RVLM neurons are more active in rats with chronic heart failure (CHF) compared to sham rats (10). Wang *et al.* (2009) reported that glutamate receptors, NMDA and non-NMDA in RVLM are involved in increasing tonic activity of the sympathetic nerves in CHF (41). Although our study was conducted on healthy rats, high plasma levels of resistin have a direct relationship with cardiovascular diseases such as CHF (33) and hypertension (42). As we know, sympathetic nerve activity increases in these conditions (30, 31). Moreover, it was reported that central administration of resistin into lateral ventricle increases SNA (43) via phosphatidylinositol 3-kinase (34). Thus, the results of this study provide a new insight to understand the relationship between the activity of sympathetic nervous system and cardiovascular diseases when plasma levels of resistin are high. 

The results of this study also showed that injection of CRF into RVLM increased cardiovascular responses (AP and HR), whereas cardiovascular responses produced by resistin into PVN decreased by astressin injected into RVLM, which was in agreement with the studies of Milner *et al.* (1993) and Bardgett *et al*. (2014). The results also indicated that injection of astressin along with AP5 can decrease cardiovascular responses produced by resistin injection into PVN. Many studies have shown that CRF type-1 receptor exists in cellular bodies and dendrites of many neurons in the nuclei regulating cardiovascular activity, such as PVN and RVLM (13, 44). Milner *et al*. (1993) stated that “bilateral injections of CRF in a dose-dependent manner into RVLM of urethane-anesthetized rats showed a dose-related increase in AP and HR” (12). Bardgett *et al*. (2014) indicated that the activity of lumbar and splanchnic sympathetic nerves increased by the activation of CRF receptors within RVLM via the activation of descending inputs from PVN (12). In addition, it was suggested that more than 30% of PVN neurons that projected to RVLM express CRF as a co-neurotransmitter (11). Interestingly, in our study the injection site for resistin was medial parvocellular part of PVN, which provided a major source of CRF input to RVLM (12, 45). Therefore, it is possible that the injection of resistin into different part of PVN could act as a stimulator to release CRF, which could stimulate RVLM sympathoexcitatory neurons and lead to the increase of AP and HR (10, 12). On the other hand, the results of this study showed that the depressor response generated by astressin+ AP5 injected into RVLM were significantly stronger than the depressor response generated by astressin or AP5 injected into RVLM, following the injection of resistin into PVN. The depressor response generated by AP5 injected into RVLM was significantly stronger than the depressor responses generated by astressin injected into RVLM, following the injection of resistin into PVN. Therefore, CRF may act as a co-neurotransmitter along with glutamate in the excitatory pathway from PVN to RVLM with an important role in synaptic excitatory of sympathoexcitatory neurons in RVLM. Furthermore, it was reported that a portion of pressor responses induced by PVN may be mediated by neural pathways, which do not include any components of RVLM (4). 

The results of this study indicated that injection of ANG II into RVLM could produce cardiovascular responses, while injection of losartan into RVLM could somewhat reduce cardiovascular responses produced by resistin injection into PVN. It was reported that the pressor and depressor responses induced by injection of ANG II and losartan into RVLM, respectively, were mediated by AT1R (15, 46-48). The AT1Rs exist in cellular bodies and axonal terminals of many neurons in the nuclei regulating cardiovascular activity, such as PVN, supraoptic nucleus (SON), and RVLM (15, 46-48). ANG II by the activation of cellular signaling pathways of AT1Rs can increase glutamate release in RVLM and inhibit GABA release in RVLM and IML (49). According to our results, cardiovascular responses to ANG II injected into RVLM were significantly greater and longer than L-glutamate or CRF; it is possible that the long-term effect of ANG II injected into RVLM is due to intracellular signaling pathways, which occur via a Gs protein dependent mechanism and increase reactive oxygen species. Previous studies showed that injection of antagonists of either angiotensin receptors or glutamate receptors into RVLM reduces HR and AP (15, 46, 47). Moreover, Tagawa and Dampney (1999) discussed that cardiovascular response produced by injection of bicuculline into PVN is reduced by inhibiting AT1 receptor by 40-50% of the initial response (48). They showed that the responses were not altered by blockage of glutamate receptors in RVLM (48). In another study, Tagawa *et al*. (1999) indicated that blockage of AT1 receptor reduced the activation of RVLM sympathoexcitatory neurons via a mechanism independent of glutamatergic or GABAergic neurotransmission (50). Based on the results of these studies, there is an agreement that angiotensinogenic transmission within RVLM is independent of other neuronal mechanisms such as GABA and glutamate, which regulate the activity of sympathetic nervous system, AP and HR. In accordance with these evidences, the results of this study showed that the depressor response produced by injection of AP5+losartan or astressin +losartan into RVLM was significantly greater and longer than the depressor response generated by astressin+ AP5 or even losartan, astressin or AP5 injected into RVLM, following the injection of resistin into PVN. Also, the depressor response generated by astressin+ AP5 injected into RVLM was significantly lower than the depressor response generated by AP5+losartan or astressin +losartan. Therefore, according to our results, it is possible that the cardiovascular responses produced by injection of resistin into PVN may be mediated by other neuronal transmissions such as glutamatergic and CRFergic neurotransmissions. 

## Conclusion

To sum up, such data suggested that 1) the PVN could be one of the main sites for actions of resistin. 2) The angiotensinogenic transmission within RVLM is independent of other neuronal transmission such as glutamate and CRF, which also regulates AP and HR. 3). It is possible that the cardiovascular responses produced by injection of resistin into PVN may be mediated by glutamatergic and CRFergic neurotransmissions within RVLM. The results provide a new and potentially important insight into neuronal mechanisms when plasma resistin levels are high. Moreover, neurotransmitters that can influence the activity of PVN-RVLM neurons can be used as therapeutic targets in cardiovascular diseases such as HF and hypertension, which are associated with high levels of resistin.
